# Every reason to discontinue lithium

**DOI:** 10.1186/s40345-014-0012-y

**Published:** 2014-11-08

**Authors:** Manuel E Fuentes Salgado, Bruce Sutor, Robert C Albright, Mark A Frye

**Affiliations:** Department of Psychiatry and Psychology, Mayo Clinic Depression Center, Rochester, MN 55901 USA; Department of Internal Medicine, Division of Nephrology, Mayo Clinic, Rochester, MN 55901 USA; Department of Psychiatry, Facultad de Medicina Clínica Allemana, Universidad del Desarrollo, Vitacura 5951, Comuna de Vitacura, Santiago, Chile

**Keywords:** Lithium, Toxicity, Efficacy

## Abstract

Lithium as a gold standard therapy for bipolar disorder is well known to have a number of medical comorbidities that impact renal, parathyroid, and thyroid function. Despite these medical comorbidities, there remains a group of lithium-responsive lithium-treated patients who have maintained mood stability for decades. The risk/benefit ratio of end organ toxicity/mood stability must be evaluated in each individual case.

## Correspondence

Lithium is the gold standard therapy for bipolar disorder, treating acute mania and depression, preventing episode recurrence, and reducing suicide risk (Cipriani et al. 2005; Geddes et al. 2004). Because of its side effect burden, narrow therapeutic window, and an increasing pharmacopeia, there are clinical situations in which lithium is no longer a first line treatment and/or when toxicity necessitates drug discontinuation (Malhi et al. 2012; McKnight et al. 2012).

Lithium has an acute (nephrogenic diabetes insipidus) and chronic (interstitial fibrosis, segmental glomerulosclerosis, and/or tubulointerstitial changes associated with renal insufficiency) effect on the kidney (Baig et al. 2011; Bassilios et al. 2008; Grunfeld and Rossier 2009). Lithium interferes with thyroid metabolism and increases the incidence of overt and subclinical hypothyroidism and may cause hyperparathyroidism with a high incidence of multiglandular disease (Hundley et al. 2005; Kleiner et al. 1999). Renal, thyroid, and parathyroid toxicity related to long-term treatment often contribute to drug discontinuation (Hundley et al. 2005; Kleiner et al. 1999; McKnight et al. 2012). We report a case of lithium-responsive bipolar disorder and the risk/benefit ratio of ongoing mood stability vs. end organ toxicity.

Ms. D is a 60-year-old woman with bipolar I disorder. Her early course of illness was characterized by two hospitalizations in 1982 for major depression. Her only hospitalization for euphoric mania was in 1983, at which time lithium was started. After mood stability was achieved, the medication was discontinued, with recurrence of major depression several months later. Restarting lithium, she noted mood improvement, maintained lithium 1,200 mg for another 3 years (1985 to 1988) before discontinuing the drug. For a 1996 postpartum depression unresponsive to divalproex sodium, she returned to lithium and again achieved mood stability (1997 to 2001). There was no family history of bipolar disorder or previous use of lithium.

In August 2001, she was diagnosed with a retroperitoneal myxofibrosarcoma with kidney involvement and underwent a right nephrectomy. Adjustment to her lithium dose was done to maintain levels at 0.6 to 0.8 mmol/L. Her serum creatinine nonetheless slowly began to increase, with a 1.5 mg/dL peak 10 years later in 2011. The diagnostic work-up included a creatinine clearance (42 mL/min) and a computerized tomography scan that identified small cysts. Stage 3 chronic renal insufficiency and chronic interstitial nephritis of her left solitary kidney was diagnosed and after nearly 15 years of non-continuous lithium treatment (three trial periods) the drug was discontinued.

Within 1 week of starting carbamazepine, she developed a severe vaginal and perineal rash. The drug was discontinued, and treatment was switched to maintenance risperidone 1 mg. After 5 months of treatment, symptoms of major depression emerged. She was titrated to a quetiapine dose of 100 mg but complained of excessive sedation and worsening depression. In 2011, lithium was reintroduced and remains today, dosed at 300 to 450 mg maintaining levels between 0.4 and 0.6 mmol/L. There was consideration of a lamotrigine trial, but she declined due to the previous adverse reaction of other anticonvulsants and her insistence upon returning to lithium, which she found helpful.

Over time, her creatinine/estimated glomerular filtration rate has progressed from 1.5/36 in 2009 to 1.8/29 in 2014. Since 2011, her thyroid-stimulating hormone has fluctuated between subclinical hypothyroidism (thyroid-stimulating hormone 6.8 mIU/L) and transient subclinical hyperthyroidism (0.01) status postparathyroidectomy; she has never been on long-term levothyroxine replacement. In 2012, she was hospitalized for a sudden abdominal pain secondary to a cecal volvulus that was resolved by ileocecectomy. During the hospitalization, she developed asymptomatic hypernatremia probably secondary to nephrogenic diabetes insipidus related to lithium therapy. In 2013, mild hypercalcemia was identified with further testing revealing primary hyperparathyroidism; calcium supplement was discontinued and a partial parathyroidectomy was performed. Subsequent calcium levels have remained slightly elevated, without evidence for metabolic or surgically active urolithiasis.

### Discussion

There remains a group of lithium-treated patients who have maintained decades of mood stability and who, when the drug is discontinued for side effect toxicity, develop significant mood destabilization. The risk/benefit of end organ toxicity/mood stability must be evaluated in each individual case. Up to 40% of the patients who received long-term lithium experienced nephrogenic diabetes insipidus; whereas the risk of end-stage renal failure is greater than in healthy controls, the absolute risk is low approximately 0.5% (Baig et al. 2011; McKnight et al. 2012). The benefit of discontinuing lithium once the kidney begins to fail is a controversial decision. There are no clear guidelines about when and how to stop the prescription. While not in this case, the beneficial renal effect of discontinuing lithium is observed mainly in patients with moderate chronic kidney disease (creatinine clearance >40 mL/min) (Baig et al. 2011; McKnight et al. 2012). Moreover, recent research from a Swedish renal registry suggests that more modern treatment principles of lithium maintenance (i.e., serum levels 0.5 to 0.8 mmol/L vs. 0.8 to 1.2 mmol/L, regular and frequent renal function monitoring) may have reduced this lithium-associated renal event (Aiff et al. 2014). The rate of hypothyroidism and primary hyperparathyroidism is increased sixfold and tenfold, respectively, related to lithium treatment (McKnight et al. 2012). There are no clear recommendations about the threshold for initiation of thyroid supplementation in lithium-treated patients with subclinical hypothyroidism. However, it is increasingly recognized that subtle change in thyroid-stimulating hormone and free thyroxine are associated with rapid cycling and bipolar depression recurrence (Frye et al. 1999; Frye et al. 2009). Lithium can be associated with hypercalcemia more commonly than hyperparathyroidism (Lally et al. 2013) and can exacerbate preexisting hyperparathyroidism, increasing the rate of multiglandular disease; subtotal parathyroidectomy, intraoperative parathyroid hormone determination-guided excision, or the use of calcimimetics are the proposed treatment options (Szalat et al. 2009). In this case, after subtotal parathyroidectomy, the serum calcium levels remained elevated (parathyroid hormone normal), which is not the usual response in the lithium-related cases. In the final overview of lithium-related toxicity, it is important to differentiate between side effects due to inappropriate use of the drug (from the standpoint of both overdose or dehydration) and practice evolution of lithium maintenance treatment monitoring (Aiff et al. 2014). Clinical assessment for appropriateness of lithium treatment selection (i.e., response predictors) and close clinical monitoring may decrease the risk/benefit ratio.

### Conclusion

The patient's decision to always return to lithium was based on the recognition of superior mood stabilization over other treatments, support and validation from her family regarding treatment benefit of lithium, and access to close medical follow-up. Her lithium course of illness response was remarkable for several reasons. First, she had clear response predictors (i.e., euphoric mood, absence of substance abuse, non-rapid cycling) with evidence of drug discontinuation three times with depressive episode recurrence, and, more notably, re-responsive illness with lithium reintroduction. Second, the patient had either poor tolerability (quetiapine), adverse allergic reaction (carbamazepine), or non-response (risperidone, divalproex sodium) to other treatment options. Her multidisciplinary treatment including psychiatry and nephrology teams tried to use other mood stabilizers that could help her to stabilize her mood and not deteriorate her physical health, but every other treatment failed for lack of efficacy or poor tolerability. In spite of her medical illness burden (status postnephrectomy, stage 3 renal failure, nephrogenic diabetes insipidus, status posthyperparathyroid resection with persistent hypercalcemia, history of subclinical hypothyroidism, and hyperthyroidism), lithium continued to be her maintenance mood stabilizer. The patient had every reason to discontinue lithium, but in her opinion, guided by her medical providers, lithium provided the best mood stabilization for management of bipolar disorder.

## Abbreviations

Mg: milligram(s)
mg/dL: milligram(s) per deciliter
mIU/L: milli-international units per liter
mL/min: milliliter(s) per minute
mmol/L: millimole(s) per liter
